# Family resilience in patients with gynecological malignant tumors after radical hysterectomy: based on the Walsh family resilience framework

**DOI:** 10.3389/fonc.2025.1522237

**Published:** 2025-04-04

**Authors:** Xinru Zhang, Xi Li, Xindi Wang, Yiteng Chen, Yu Guo, Weiqing Ruan

**Affiliations:** ^1^ Nanfang Hospital, Southern Medical University, Guangzhou, Guangdong, China; ^2^ School of Nursing, Southern Medical University, Guangzhou, Guangdong, China

**Keywords:** gynecological malignant tumors, radical hysterectomy, family resilience, the Walsh family resilience framework, qualitative research

## Abstract

**Aim:**

Explore and analyze the family resilience of patients with gynecological malignancies after radical hysterectomy, providing a theoretical basis for the formulation of future intervention measures.

**Methods:**

Using a phenomenological descriptive qualitative research method, 17 patients who underwent radical surgery for gynecological malignancies were selected for semi-structured interviews. Data analysis and theme extraction were conducted using Colaizzi data analysis method and NVivo V.12.

**Results:**

Three themes and eight sub-themes were extracted: family belief system (confront surgical challenges head-on, attribute positive significance to adversity, stay positive), family organization model (timely adjustment of family roles, family cohesion, get support and help from others), and family communication and problem solving skills (communicate to eliminate negative emotions, collaborative problem solving).

**Conclusion:**

This study indicates that the family belief system is the solid foundation of family resilience, the family organizational pattern serves as a buffer when the family faces adversity, and positive communication and collaborative problem solving create a positive feedback loop that enhances family resilience. Future interventions could enhance patients’ family resilience from the perspective of family strengths.

## 1 Introduction

Family resilience refers to a psychological characteristic that helps family members cope positively and maintain stable family functioning when facing stress, crises, and challenges (1). With the advancement of positive psychology research (2, 3), family resilience has gradually become an important factor in psychological interventions for cancer patients, especially female cancer patients. In recent years, the incidence of gynecological malignancies has been increasing annually, and the age of onset has been gradually becoming younger, particularly for cervical cancer, endometrial cancer, and ovarian cancer (4). Radical hysterectomy is the standard treatment for patients with early-stage gynecological malignancies (5, 6). However, this treatment often comes with the loss of sexual and reproductive functions, leading to psychological issues such as anxiety, depression, sleep disturbances, and post-traumatic stress disorder (7–10). These psychological problems not only severely impact patients’ postoperative recovery and quality of life but also disrupt and damage patients’ family functioning (11, 12).

In the pathway to alleviating patients’ psychological burdens, family resilience plays a crucial regulatory role (13). Previous studies have shown that when family members collectively experience the diagnosis and treatment of cancer, family resilience can help maintain the stability of family structure and normal functioning by promoting positive coping and constructive communication, thereby assisting both patients and families in overcoming difficulties together (14, 15). A cross-sectional study on the meaning of life in breast cancer patients indicates that the level of family resilience is significantly positively correlated with their sense of life’s meaning (16). Patients with higher levels of family resilience are better able to see the positive significance of adverse events. This demonstrates that family resilience, as an important protective stress resource, plays a crucial role in helping patients and their families better adapt to the challenges and trauma brought on by cancer treatment.

Unlike the many studies conducted by scholars on breast cancer or pediatric cancer populations (17–20), there is relatively little research on family resilience in the postoperative population of gynecological malignancies. A quantitative study on cancer patients in South Korea shows that regardless of the type of cancer, positive psychological factors such as self-efficacy, hope levels, and self-esteem are significantly positively correlated with family resilience (21). In a cross-sectional study of Chinese gynecological cancer patients, it was found that their level of family resilience is relatively low, indicating significant potential for improvement (22). The family resilience of patients after radical surgery for gynecological malignancies differs significantly from that of other populations. Patients undergoing radical hysterectomy not only need to undergo postoperative rehabilitation for 3 to 6 months, but also face the absence of the uterus, a characteristic female organ, can lead to more complex negative emotions such as anxiety and depression in this already sensitive group of women (7–9). Therefore, it is crucial to focus on the family resilience experiences of patients after radical surgery for gynecological malignancies from the patient’s perspective. However, to our knowledge, there are currently no similar studies.

In the field of family resilience, the Walsh family resilience framework is the most explanatory theoretical framework (23). Based on family systems theory, this framework views the family as an independent functional unit. Walsh (23) believes that family resilience is a process of interaction between individuals, families, and the external environment, and summarizes the formation of family resilience into three main areas: family belief system, family organization model, and family communication processes. These three areas coordinate with each other to help families cope with stress and challenges. This framework has been successfully applied to lung cancer patients (15), dementia patients (24), schizophrenia patients (25), bereaved family support (26), and others. Therefore, the purpose of this study is to describe family resilience of patients after radical surgery for gynecological malignancies based on the Walsh family resilience framework. We hope that the results of this study will enrich the theory of family resilience from the perspective of gynecological malignancies and provide a theoretical basis for formulating family-centered early intervention measures. This, in turn, will further enhance family resilience and improve the prognosis and overall family outcomes of patients after radical surgery for gynecological malignancies.

## 2 Methods

### 2.1 Study design and setting

This study was conducted in the gynecology ward of a tertiary hospital in Guangzhou, China. Patients with gynecological malignancies who undergo radical hysterectomy typically spend 3 to 5 days in the ward for postoperative recovery. Therefore, the investigation period for this study was set for one week after surgery, when patients’ conditions had stabilized. A phenomenological descriptive qualitative research method was employed to report the cross-sectional results of patients with gynecological malignancies following radical surgery. This study was based on the Walsh family resilience framework, which guided the interview outline and data analysis of this research. This study strictly adhered to the consolidated criteria for reporting qualitative research (COREQ) guidelines (27).

### 2.2 Participants

Using purposive sampling to recruit research subjects. Given the cultural significance of family relationships in Chinese society, we intentionally included participants with diverse family structures. The inclusion criteria are as follows:

1. Patients who are newly diagnosed with gynecological malignancies and have undergone radical surgery treatment;
2. Aged ≥18 years;
3. Clear consciousness, no language communication barriers;
4. Informed consent, voluntarily participating in this study.

Exclusion criteria are as follows:

1. Those with severe mental illness or cognitive impairment;
2. Those with other types of tumors.

Data collection ceased when data saturation was reached. A total of 17 eligible patients were invited to participate in this study, and none declined.

### 2.3 Data collection

Semi-structured interviews were conducted in July 2024. Firstly, two researchers systematically reviewed the demographic and clinical data of patients on the day they confirmed the need for a radical hysterectomy. At the same time, in order to gain a deeper understanding of patients’ physical and mental status as well as their family situation, the researchers engaged in in-depth discussions with patients’ attending physicians and responsible nurses. To maximize the diversity of the sample, the researchers integrated clinical data and feedback from medical staff to identify potential participants, ensuring that the participants had a diverse social demographic background with varying family structures, diagnoses, and ages. After identifying potential participants, an additional researcher invited them to participate in the study. Those who agreed to participate signed a formal informed consent form.

The interviews for this study were conducted one week post-surgery, with each patient receiving a face-to-face in-depth interview in the private patient interview room of the gynecology ward. No prior relationships existed between the researchers and the patients before this study commenced. The day before the interviews, nurses introduced the interviewer to the participants. The interviewer then coordinated the interview schedule with the patients and established a positive rapport. The interviewer followed the interview outline and used a recorder and notebook to document the participants’ tone, facial expressions, and emotions. The interview outline was designed based on the Walsh family resilience framework and was revised during pre-interviews with the first three participants (as shown in **Table 1**). The interviewer listened attentively during the interview, avoided inducing or judging, and encouraged the interviewee to fully express themselves. Each interview lasted 20-40 minutes. To ensure reflection, the interviewer reflected on the interview results and wrote a reflection diary after each interview, adjusting their tone for the next interview to improve efficiency.

**Table 1:** Interview outline for patients after radical surgery for gynecological malignancies.

Patients after radical surgery for gynecological malignant tumors
1. When the doctor tells you that you need to undergo radical surgery, how do you and your family feel?
2. After undergoing radical surgery, what changes have occurred in your life and family? What are your thoughts on these changes?
3. During the surgical treatment phase, what difficulties did you and your family encounter? How did you and your family cope with these difficulties?
4. After undergoing radical treatment, have you received help from relatives, friends, or others? What specific areas did they assist you with?
5. What areas do you still need help with?

### 2.4 Data analysis

Using NVivo V.12 software for data organization and analysis. After each interview, the researcher conducting the interview organized the interview data within 24 hours to promptly adjust the interview strategy. Two researchers independently analyzed, summarized, extracted, and integrated the themes of the text materials using Colaizzi’s 7-step method, which includes the following steps: (1) carefully read the data in conjunction with the interview diary, interpret the meaning of the data, and explain non-verbal dialogues word by word; (2) analyze the data verbatim and extract feature data highly relevant to the Walsh family resilience framework, such as family belief systems, communication patterns among family members, and social support systems; (3) code the recurring themes related to the Walsh family resilience framework; (4) gather the coded viewpoints to form preliminary themes; (5) determine the essence and details of each theme, and generate clear definitions and names for each theme, referencing the Walsh family resilience framework; (6) repeatedly compare similar themes to elevate the main theme; (7) return the theme structure to the interviewee for verification.

### 2.5 Rigor and reflexivity

All researchers received comprehensive training in qualitative research courses. A literature review in the field of family resilience was conducted before the research design to better understand the connotation of family resilience. During the interview, a clinical nurse specialist with expertise in mental health care was on standby outside the interview room to promptly address any emotional issues that may arise with the patient. During data analysis, ‘investigator triangulation’ was included in the analysis strategy to reduce potential biases from a single analytical perspective. To determine whether sufficient data had been collected, the researchers reviewed the study objectives, data richness, and analysis methods (28). After discussing the analysis results and reaching a consensus, the results were sent to the participants for confirmation to avoid researcher bias.

### 2.6 Ethical considerations

This study has received ethical approval from the research ethics committee (Decision number: NFEC-2024-479). All patients were informed about the purpose of the study, the data collection process, the benefits and risks of participation, confidentiality, and other relevant information before the study began, and they signed an informed consent form. We ensured that patients knew they had the right to terminate the interview at any time if they experienced any discomfort during the process. Throughout the interview, the interviewer adjusted the pace and duration based on patients’ emotional responses and rescheduled the interview if necessary. During data analysis, researchers strictly adhered to the principles of anonymity and confidentiality. After the interview, we provided patients with the contact information of the hospital’s psychological experts, so they could reach out at any time if they experienced any psychological distress during their postoperative recovery.

## 3 Results

### 3.1 Participant characteristics

A total of 17 patients with gynecological malignancies who underwent radical hysterectomy participated in this study. The average age of the participants was 47.11 years, including 7 case of cervical cancer, 4 case of ovarian cancer, and 6 case of endometrial cancer. Among them, 8 patients were in stage I of cancer, 7 were employed, and 15 were married. Detailed information was shown in **Table 2**.

**Table 2:** Demographic characteristics and disease information of 17 participants.

Variables	Number	Frequency (%)	Mean ± SD
Age			47.11 ± 10.23
Type of cancer			
Cervical cancer	7	41.18	
Ovarian cancer	4	23.53	
Endometrial cancer	6	35.29	
Cancer staging			
I	8	47.06	
II	6	35.29	
III	3	17.65	
Educational status			
Elementary school and below	8	47.06	
Junior high school	1	5.88	
High school	4	23.53	
Undergraduate	4	23.53	
Employment status			
Employed	7	41.18	
Unemployed	10	58.82	
Marital status			
Widowed	2	11.76	
Married	15	88.24	
Number of children			
0ne	4	23.53	
Two	7	41.18	
Three	4	23.53	
Four	2	11.76	

### 3.2 Themes

The data analysis process was based on the Walsh family resilience framework (23), and three main themes were identified: family belief system, family organization model, and family communication and problem solving skills. These themes were further divided into eight sub-themes: confront surgical challenges head-on, attribute positive significance to adversity, stay positive, timely adjustment of family roles, family cohesion, get support and help from others, communicate to eliminate negative emotions and collaborative problem solving. The example quotation was shown in **Table 3**.

**Table 3:** Example quotations of themes for family resilience.

Theme	Sub-theme	Example quotations
Family belief system	confront surgical challenges head-on	First, I had to face this surgery myself. Although it couldn’t guarantee a 100% cure, there was at least a 50% chance. (N1)
		Anyway, the doctor said to do it, so I did. I was not like before when I had been timid. Now I’m fine, and I’m not afraid of anything. (N7)
		I was not afraid, I just wanted to find the best treatment plan. (N15)
	attribute positive significance to adversity	This time, a check-up revealed that I actually had cancer. This was my body’s warning, telling me that I hadn’t been taking care of it properly. (N4)
		The total hysterectomy was actually a beneficial experience for me. After going through this, I will cherish my body more and pay more attention to health check-ups. (N5)
		My daughter told me that the surgery was not a bad thing, and that being able to have the surgery was already a great blessing. This made me realize that surgery is not a terrible thing. (N8) To some extent, I am very grateful for this surgery. It made me, a workaholic, start paying attention to changes in my body. (N15)
		Being able to have surgery is a good thing, right? It means I can still be treated, doesn’t it? (N16)
		After this, my husband knows how to care for me more, and the child is more obedient too. I even have to thank the surgery, hahaha. (N17)
	stay positive	My daughter was very optimistic, always smiling next to me every day, and I was influenced by her. (N8)
		My personality is inherently good, and I always try to think positively about everything that happens. (N9)
		When relatives asked me about my illness, I told them it was like having a polyp removed. For me, it was just like having a polyp removed. (N11)
		My mindset is very positive. I need to find ways to treat it. Being disappointed is useless, and my mood can affect the treatment outcome. (N15)
		I think it is all about mindset. A good mindset can solve many problems. (N16)
Family organization model	timely adjustment of family roles	I have two children at home. My daughter has been with me since my surgery, and my son stayed with me for a week. I feel very happy. (N4)
		My two grandsons are on summer vacation now. I used to take care of them, but now my daughter-in-law is taking care of them, allowing me to focus on my treatment. (N5)
		When the kids were small, I used to comfort them. Now they comfort me, making me relax every day as if I were a child. (N7)
		My husband never did housework before, but after I was hospitalized, he took care of all the household chores. (N17)
	family cohesion	The meticulous care from my family had given me great comfort and confidence to overcome the illness, which made me feel like I haven’t been abandoned. (N1)
		What touched me the most was my mother-in-law. After I fell ill, she took care of me as if I were her own daughter and often told me not to think about preserving my uterus, and if the doctor recommended removal, then we should proceed with it. (N3)
		After my daughter found out that I had cancer, she immediately searched Baidu and other online resources, telling me that this is early-stage and the chances of recovery are very high. She also said, ‘Mom, don’t worry, I’ll always be with you’. (N4) Thinking about lying on the operating table made me scared, but thinking about how my children would have had no mother to rely on if I had passed away made me brave enough to face it. (N6)
		My husband has always been fighting alongside me, both physically and mentally. He can handle it better. (Crying) (N13)
		Every time I felt down, I look at videos and photos of my daughter on my phone. Seeing her smile and hearing her call me ‘Mom’ filled me with strength, as if no illness could ever defeat me. (N14)
		Having family makes me feel like there’s someone supporting me from behind. (N14)
	get support and help from others	After I got sick, my boss was very concerned. Once I handed over my work, I no longer had to worry about work matters and could focus on my treatment. (N1)
		The hospital food was quite bland, and I didn’t have much of an appetite. To make me eat more, my nephew ran all over the hospital to find me something tasty (laughs). (N2)
		After learning about my cancer, I immediately contacted a colleague in the same situation. She shared her medical and recovery experiences with me. (N12)
		I have a very opinionated best friend who helped me make various decisions throughout the process, and the company leaders and colleagues were also very concerned. (N15)
		The doctor was very professional and often explained things I didn’t understand, which made me feel very reassured. (N17)
Family communication and problem solving skills	communicate to eliminate negative emotions	When I couldn’t sleep in the hospital, I called my husband to wake him up, hahaha, and chatted with him intermittently until I fell asleep again. (N2)
		When I learned about my need for a total hysterectomy at home, I was very sad at the time. But after venting to my husband, I accepted it. (N3)
		At first, I was very resistant to signing the surgical consent form. I felt that once I signed it, the uterus, which is a symbol of femininity, would no longer be a part of me. The risks and complications of the surgery also frightened me a great deal. Later, my parents rushed to the hospital overnight and talked with me for a long time. I felt like I had a lot more people behind me. I realized that being healthy and alive is the most important thing, so I signed the surgery consent form myself. (N17)
	collaborative problem solving	Those days, I cried every day. My eldest daughter searched on her phone for which hospital was good and which doctor was an expert in this field. After finding the right one, she hurriedly brought me there, telling me that even if she had to borrow money, she would make sure I got treated. (N6)
		This made me feel very happy and more willing to put down my phone and chat with them.(N8)
		After being hospitalized, my parents, old friends, and former colleagues all helped me find doctors and solutions. (N12)
		I come from a single-parent family. After being hospitalized, my daughter had no one to take care of her, so my mom and older sister took turns caring for her. (N15)

#### 3.2.1 Theme 1: family belief system

A positive family belief system is the core of family functioning and a strong foundation for cultivating family resilience. Beliefs influence cognition and guide actions, and people’s understanding of themselves and their experiences is based on their beliefs (23). Beliefs and actions mutually influence each other, actions and their consequences reinforce people’s beliefs. When facing surgical challenges, confronting them directly and attributing positive meaning to the adversity, while maintaining an optimistic attitude, can help the entire family smoothly navigate the surgical period and enhance the family’s ability to cope with challenges.

##### 3.2.1.1 Subtheme 1: confront surgical challenges head-on

When it came to surgical challenges, almost all patients believed that they should face the surgery head-on, as escaping could not solve the problem.

‘First, I had to face this surgery myself. Although it couldn’t guarantee a 100% cure, there was at least a 50% chance.’ (N1)

‘Anyway, the doctor said to do it, so I did. I wasn’t like before when I had been timid. Now I’m fine, and I’m not afraid of anything.’ (N7)

‘I was not afraid, I just wanted to find the best treatment plan.’ (N15)

##### 3.2.1.2 Subtheme 2: attribute positive significance to adversity

The uncertainty during the perioperative period and postoperative recovery caused patients to experience fluctuating emotions. However, they still saw a positive side in adversity, and some patients said that the surgery was not necessarily a bad thing.

‘The total hysterectomy was actually a beneficial experience for me. After going through this, I will cherish my body more and pay more attention to health check-ups.’ (N5)

‘My daughter told me that the surgery was not a bad thing, and that being able to have the surgery was already a great blessing. This made me realize that surgery is not a terrible thing.’ (N8)

‘To some extent, I am very grateful for this surgery. It made me, a workaholic, start paying attention to changes in my body.’ (N15)

‘After this, my husband knows how to care for me more, and the child is more obedient too. I even have to thank the surgery, hahaha.’ (N17)

##### 3.2.1.3 Subtheme 3: stay positive

Maintaining a strong and optimistic attitude when facing surgical challenges can lead to better coping with illness and is of great significance for improving the family atmosphere and establishing a family belief system.

‘My daughter was very optimistic, always smiling next to me every day, and I was influenced by her.’ (N8)

‘My personality is inherently good, and I always try to think positively about everything that happens.’ (N9)

‘My mindset is very positive. I need to find ways to treat it. Being disappointed is useless, and my mood can affect the treatment outcome.’ (N15)

‘I think it is all about mindset. A good mindset can solve many problems.’ (N16)

#### 3.2.2 Theme 2: family organization model

After undergoing a radical hysterectomy, it was inevitable that the original family structure of patients with gynecological malignancies would be affected. A flexible family organization model serves as a buffer when the family is impacted. When facing adversity, the family must readjust its structure based on the actual situation and mobilize internal and external resources to alleviate pressure in order to effectively handle crises or adversity. A flexible family organization model includes timely adjustment of family roles, family cohesion, and getting support and help from others.

##### 3.2.2.1 Subtheme 1: timely adjustment of family roles

The patient, after undergoing surgery and being hospitalized, was unable to fulfill their previous family role, leading to an adjustment in the division of roles among family members to maintain the normal functioning of the family.

‘My two grandsons are on summer vacation now. I used to take care of them, but now my daughter-in-law is taking care of them, allowing me to focus on my treatment.’ (N5)

‘When the kids were small, I used to comfort them. Now they comfort me, making me relax every day as if I were a child.’ (N7)

‘My husband never did housework before. But after I was hospitalized, he took care of all the household chores. ‘ (N17)

##### 3.2.2.2 Subtheme 2: family cohesion

Cohesion is the emotional connection among family members. Families with strong cohesion support and rely on each other to collectively resist external risks.

‘The meticulous care from my family had given me great comfort and confidence to overcome the illness, which made me feel like I haven’t been abandoned.’ (N1)

‘What touched me the most was my mother-in-law. After I fell ill, she took care of me as if I were her own daughter and often told me not to think about preserving my uterus, and if the doctor recommended removal, then we should proceed with it.’ (N3)

‘Thinking about lying on the operating table made me scared, but thinking about how my children would have had no mother to rely on if I had passed away made me brave enough to face it.’ (N6)

‘Every time I felt down, I looked at videos and photos of my daughter on my phone. Seeing her smile and hearing her call me ‘Mom’ filled me with strength, as if no illness could ever defeat me.’ (N14)

‘Having family made me feel like there’s someone supporting me from behind.’ (N14)

##### 3.2.2.3 Subtheme 3: get support and help from others

Some patients indicated that the emotional and material support from external resources provided them with comfort during their surgical treatment.

‘The hospital food was quite bland, and I didn’t have much of an appetite. To make me eat more, my nephew ran all over the hospital to find me something tasty (laughs).’ (N2)

‘After learning about my cancer, I immediately contacted a colleague in the same situation. She shared her medical and recovery experiences with me.’ (N12)

‘The doctor was very professional and often explained things I didn’t understand, which made me feel very reassured.’ (N17)

#### 3.2.3 Theme 3: family communication and problem solving skills

Communication serves various functions, including conveying beliefs, exchanging information, expressing emotions, and solving problems. Effective communication can not only alleviate patients’ negative emotions caused by surgery but also promote mutual support and cooperation among family members. Positive communication and collaborative problem-solving reflect the supportive nature of family resilience.

##### 3.2.3.1 Subtheme 1: communicate to eliminate negative emotions

When negative emotions arose due to surgery, communicating with family helped alleviate anxiety to some extent and strengthened one’s confidence in treatment.

‘When I couldn’t sleep in the hospital, I called my husband to wake him up, hahaha, and chatted with him intermittently until I fell asleep again.’ (N2)

‘When I learned about my need for a total hysterectomy at home, I was very sad at the time. But after venting to my husband, I accepted it.’ (N3)

‘At first, I was very resistant to signing the surgical consent form. I felt that once I signed it, the uterus, which is a symbol of femininity, would no longer be a part of me. The risks and complications of the surgery also frightened me a great deal. Later, my parents rushed to the hospital overnight and talked with me for a long time. I felt like I had a lot more people behind me. I realized that being healthy and alive is the most important thing, so I signed the surgery consent form myself.’ (N17)

##### 3.2.3.2 Subtheme 2: collaborative problem solving

Some patients indicated that during the surgical process, their entire family came together to collaboratively solve problems, helping them get through the challenges of the surgery.

‘Those days, I cried every day. My eldest daughter searched on her phone for which hospital was good and which doctor was an expert in this field. After finding the right one, she hurriedly brought me there, telling me that even if she had to borrow money, she would make sure I got treated.’ (N6)

‘On the day of the surgery, my sister, my older sister, my dad, and my younger brother all came. This made me feel very happy and more willing to put down my phone and chat with them.’(N8)

‘After being hospitalized, my parents, old friends, and former colleagues all helped me find doctors and solutions.’ (N12)

## 4 Discussion

Guided by the Walsh family resilience framework, this study described the family resilience of patients with gynecological malignancies after undergoing radical hysterectomy. Through the analysis of data obtained from interviews, three themes were extracted: family belief system, family organization model and family communication and problem solving skills. This study focuses on gynecological malignancies, a large patient population with long-surviving time, and particularly on, the highly traumatic experience of radical hysterectomy. It aims to enrich the research outcomes in the field of family resilience in cancer, providing researchers and healthcare professionals with a culturally specific female perspective.

Influenced by traditional Chinese family culture, family members seek support and comfort from the family belief system when facing challenges and difficulties. Positive family belief system serves as a strong foundation for cultivating family resilience. Some of the interviewees in this study stated that the optimistic attitudes of their children and husbands towards radical hysterectomy changed their own negative views, allowing them to recognize the positive impact of the surgery and maintain an optimistic outlook. This suggests that family beliefs can influence how individuals and families perceive the meaning of adversity, guiding them in making decisions and taking actions during difficult times. This finding is consistent with conclusions drawn in other studies (29–31). At the same time, the optimistic emotion can be mutually transmitted and influenced within the family, which aligns with the findings of Terrill (32) and Kuang (33). Therefore, this suggests that clinical practitioners, during the patient’s surgery, can focus on leveraging the positive influence of the patient’s family, guiding them to pay attention to the patient’s emotional changes, and leading the patient toward positive emotions and proactive coping, thereby enhancing the patient’s confidence in treatment and adherence. When designing future family resilience interventions, consider the family as a whole, implement dual interventions between patients and caregivers, rebuild positive family cognition, improve the family belief system, and lay a solid foundation for enhancing family resilience.

When adversity strikes, the family is impacted, and changes to the original family organizational structure are inevitable. The family organizational model serves as a buffer when the family is under stress, prompting the family to flexibly alter the existing model, timely adjust family roles, and demonstrate strong family cohesion (15) (34). This helps the family find a balance between stability and change, and maintain the normal operation of family functions. In this study, patients with gynecological malignancies often had to take on the responsibility of caring for their children. After they were hospitalized, family members actively took on this responsibility, demonstrating a high level of family cohesion, which allowed the patients to fully focus on their surgical treatment without any worries. When the impact is significant and the internal resources of the family are insufficient to meet the adjustment needs, obtaining support and assistance from external resources can further enhance the family’s ability to cope with the challenges of surgery (35, 36). This may include sharing experiences from fellow patients, financial support from relatives, and professional information provided by healthcare staff. This suggests that supportive therapy has significant research implications for enhancing family resilience, and existing research findings have also proven this point (37, 38). Medical institutions, communities, and relevant social organizations should emphasize the importance of external support, build a multidisciplinary, multi-level, and multi-type social support network, vigorously develop continuity of care, help gynecological cancer patients choose the best treatment plan, smoothly transition through the postoperative recovery period, and return to their families and society with a high quality of life.

Active communication and joint decision-making are positive predictors of family resilience (39). When patients face radical hysterectomy for the first time, it is understandable to experience negative emotions such as anxiety and fear due to the unknown. Multiple studies have shown that (15, 40), the self-concealment of negative emotions not only hinders the maintenance of close relationships between patients and their families but also creates gaps in emotional communication. Additionally, the accumulation of negative emotions can indirectly affect the patient’s physical condition, which is detrimental to postoperative recovery. Therefore, it is particularly important to eliminate all kinds of negative emotions generated during the operation in a timely and effective manner. Unlike most male cancer patients (40), female patients with gynecological malignancies tended to express their emotions more openly. The majority of participants in this study mentioned that when they felt depressed, they alleviated negative emotions and boosted their confidence in treatment through communication with their families. This shows that positive communication is the right coping style to eliminate bad emotions.

In addition, some participants experienced decision-making difficulties before surgery due to concerns about the effectiveness of the surgery and potential postoperative complications. Their family members provided emotional support through equal and open communication, alleviating their anxiety and helping the patients make decisions. This suggests that a good communication model can provide information support and a foundation of trust for both patients and their families, enabling joint decision-making and collaborative problem-solving. Influenced by traditional Chinese culture, personal suffering is often seen as a family matter. As a result, most participants mentioned that during surgery, the entire family would be involved in helping the patient navigate the perioperative period, which made them more willing to confide in their family members. This indicates that the collaborative problem-solving process promotes more positive communication, forming a virtuous cycle that enhances family resilience. In future intervention plans, encouraging family members to communicate openly and actively, fostering a culture of love and understanding, and enhancing the ability of family members to work together to solve problems are core elements for improving family resilience.

This study has some common limitations of qualitative research. Firstly, the research results were based on semi-structured interviews, which might have had the Hawthorne effect (41). Respondents may subjectively create a more positive and optimistic image because they are aware that they are participating in the interview. Secondly, this study was conducted in a tertiary hospital in China, which somewhat reduced the representativeness of the sample. Chinese family culture emphasizes collectivism and family responsibility, while Western cultures place more importance on individualism and self-actualization, which may lead to significant differences in family resilience. Therefore, future research could be conducted in multiple geographical regions to further explore family resilience from different cultural perspectives.

## 5 Conclusion

Based on the Walsh family resilience framework, this study delved into the family resilience of patients with gynecological malignancies after undergoing radical hysterectomy. The findings of this study suggest that the family belief system is a solid foundation for family resilience, the family organizational model is a buffer when the family faces adversity, and positive communication and collaborative problem solving form a virtuous cycle of enhancing family resilience. It is recommended that future interventions focus on the perspective of family strengths, build a positive family belief system, form a flexible family organizational model, promote effective communication and joint decision-making within the family, thereby enhancing the family resilience for patients.

## Data Availability

The raw data supporting the conclusions of this article will be made available by the authors, without undue reservation.
